# Projected return on investment of a corporate global health programme

**DOI:** 10.1186/s12889-019-7857-z

**Published:** 2019-11-07

**Authors:** Samuel Peik, Erik Schimmel, Sara Hejazi

**Affiliations:** GlaxoSmithKline, 5 Crescent Drive, Mailstop NY0200, Philadelphia, PA 19112 USA

**Keywords:** Corporate global health, Clinical preventive services, Return on investment

## Abstract

**Background:**

Health and wellbeing initiatives vary in effectiveness due to programme design and offerings. The Partnership for Prevention programme expands access to up to 40 evidence-based clinical preventive services for all employees and eligible family members as part of a unique global health initiative.

**Methods:**

Using a published RAND Europe model developed for the company, country-level return on investment was estimated over a five-year timeframe using programme utilisation data. Regional, global, and service-level averages were estimated using population-weighted country averages. Data were collected from 2012 to 2018 and analysed in 2018.

**Results:**

The programme is estimated to generate a global return of $4.28–$11.88 (after cost of investment), based on analysis of 57 countries and nearly 125,000 delivered services. Returns were positive for all regions, and immunisations, smoking cessation, and cardiovascular treatment generated the largest individual service returns.

**Conclusions:**

This global health programme is projected to generate a significant return on investment by focusing on global utilisation of clinical preventive services.

## Background

Health and wellbeing programmes are now standard among corporations and other organisations and have historically been focused on behavioural changes such as weight loss, physical activity, or other modifiable risk factors. The evidence supporting effectiveness and cost reduction for these programmes is mixed, impacted by the variation in programme offerings and execution, reliance on self-evaluation, as well as lack of consistent return or outcome measurement.

One meta-analysis estimated a $3.72 reduction in medical costs and a $2.73 reduction in absenteeism-related costs [1], while another in a specific company found a return of $1.88–3.92 [2]. Another study estimated three times higher stock prices among winners of a national employer health award compared to other firms [3]. Analysis by RAND Corporation concluded that lifestyle management aspects do not reduce healthcare utilisation or cost, noting variation in programme configuration and low participation [4]. A subsequent RAND Corporation brief with data from one employer over ten years showed per-dollar investment returns of $3.80 for disease management programmes, $0.50 for lifestyle management programmes, and $1.50 overall, noting lifestyle management programmes take longer to realise returns [5]. Another large compilation highlights programme differences and urged employers to consider the goals and organisational culture, emphasising adaptation of best practices to maximize positive results [6]. The balance of evidence suggests programmes have the potential to be effective if they are developed and implemented successfully and a longer-term outlook is used for evaluation. Execution may involve significant effort and complexity, and all programmes may not show a positive or significant return, given they are not effortless and simple solutions.

Partnership for Prevention (P4P) is a global programme that expands on existing health and wellbeing offerings and focuses on increasing access to clinical preventive services. P4P provides company employees and their benefits-eligible family members with access to up to 40 preventive healthcare services, at little to no cost, regardless of their location, job role or pay grade. The programme is an opportunity to address multiple barriers to accessing preventive healthcare such as awareness, cost, geography, and culture. This organization is the first multi-national company, to our knowledge, to develop and implement a global programme of this focus and scale. The P4P programme focuses on analysing, identifying, and addressing gaps in access to high-quality preventive services for all covered employees and dependents. Covered services are chosen based on substantial evidence of effectiveness in disease prevention or detection, based on recommendations and evidence from institutions such as the World Health Organization. The services are intended to complement, rather than duplicate, existing preventive healthcare services. This balance is accomplished by analysing current gaps and devising an individual solution for each country and market, by providing new services to fill existing gaps. In this way, P4P focuses primarily on reducing existing disparities in access to clinical preventive services.

## Methods

**Fig. 1:**
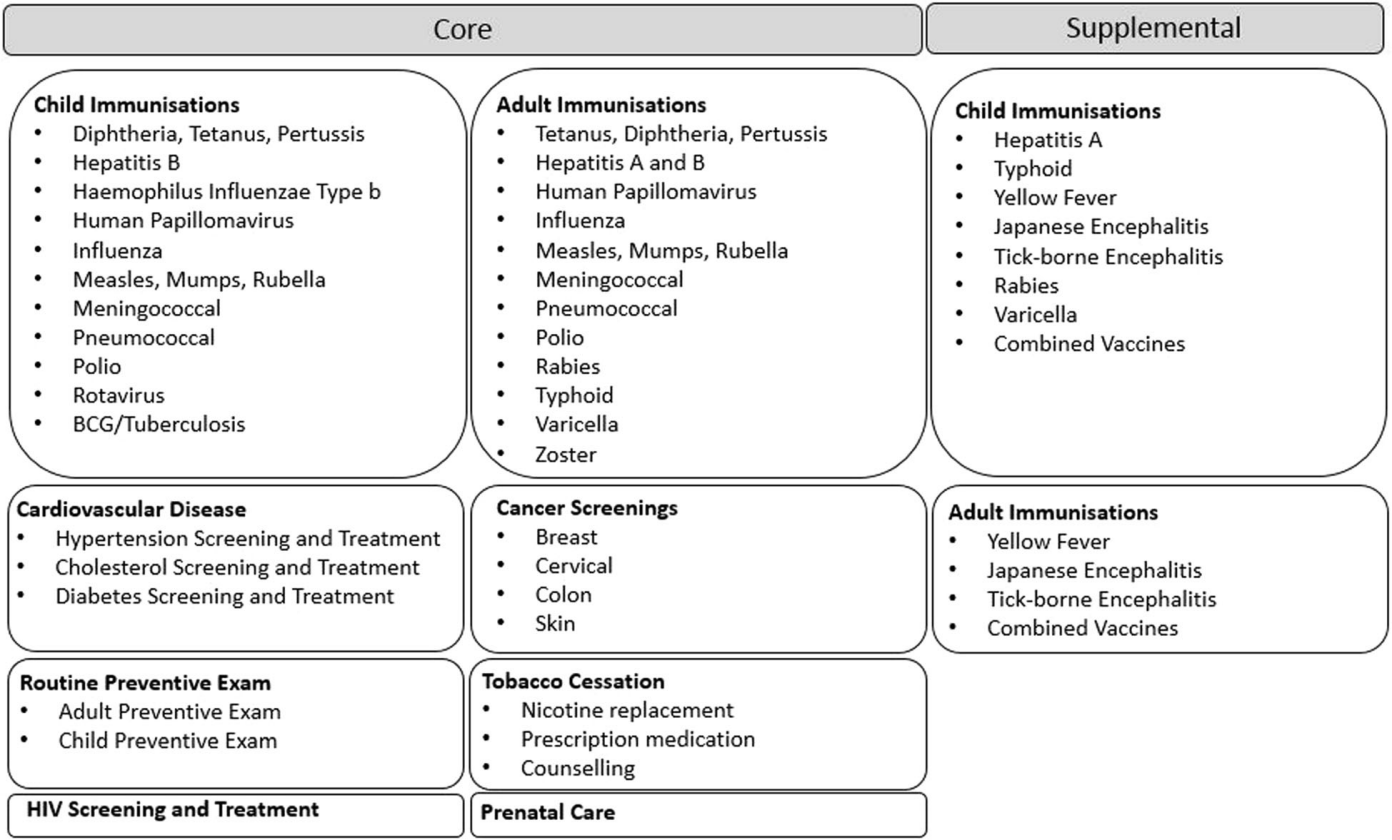
P4P Services

This study focuses on measures of effectiveness for the P4P programme, specifically return on investment (ROI) to the company, based on modelled health outcomes and actual utilisation data from individual countries for the period 2012–2018. After an initial pilot project involving four countries proved viable, the program was implemented on a regional basis with one region launching each year. P4P has collected centralised data from all active countries after program launch for the first 24 months of activity, which are then transitioned to local country management for continued execution and sustainment. P4P provides a relatively broad range of preventive services; see Fig. 1 for a full list. Core services are available to all countries, while supplemental services are applied to countries on an individual basis based on standard criteria.

### Data collection

Data are anonymised by the local P4P administrator, which is a third-party selected to deliver or manage the clinical preventive services. The administrator gives each participant a unique patient identification number. This maintains privacy while also allowing the team to analyse how many unique patients are utilising the programme in each country. Data reporting consists of descriptive information such as age and gender, and service information such as service date, type, and cost. The data were originally collected and analysed primarily to track utilisation, identify areas of improvement, and develop strategies to improve the programme locally, regionally, and globally.

### ROI model

**Fig. 2:**
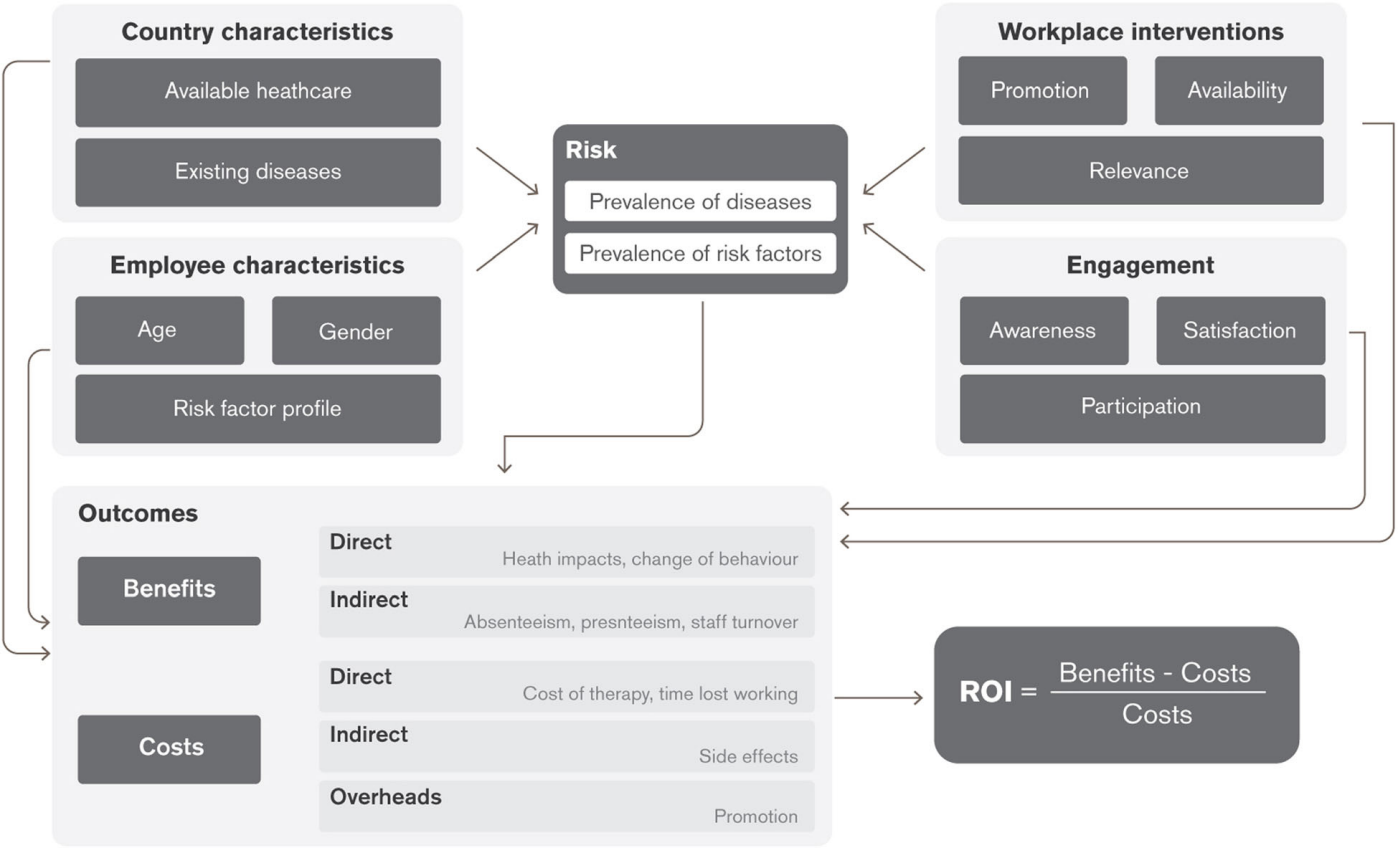
RAND Europe generalised framework for ROI assessment

The programme commissioned a report by RAND Europe [7] to evaluate the program from ROI perspective. This effort was made to expand our programme measures of effectiveness further, and to do so through an evidence-based and independent source. RAND Europe published a report in 2017 consisting of a literature review, data description, and development of a calibrated model. As a result of this effort, a specific ROI tool was created based on the case study of a single country (South Africa). This tool was designed to evaluate local utilisation data to estimate ROI on an individual country basis. (See Fig. 2 for the generalised framework utilised in developing the model).

Health outcomes were estimated based on literature and reference data collected by RAND, not individual participant health outcomes. Standard model inputs for all countries included absenteeism (due to dependent illness including risk of infection and time off), communicable disease infection risk, discount rate, and a present value of future cash flows. Additional country-specific model inputs include morbidity and mortality data (from the Global Burden of Disease database), estimated or assumed screening and vaccination rates, age and gender structure, exchange rate, workdays, programme administrative costs, employee replacement costs, time spent participating in interventions, and average daily wage. RAND Europe estimates and vetted data sources were utilised for external data to inform model assumptions, supplemented by company-specific data where required. Regional estimates were used when country-specific data was not available. Further details of the model are available in the published RAND Europe report [7].

### Data analysis

To obtain country, region, and global ROI estimations, utilisation data from the P4P programme were available through July 2018. Country estimates were performed directly by entering the data into the ROI tool. Regional and global estimates were calculated using a weighted average of ROI upper and lower bounds, weighted for each country population (also referred to as persons covered through the programme). The model initially projects over five years, which was chosen for consistency with the RAND Europe publication as well as providing a reasonable timeline for investment return to the organisation. ROI estimates were made after the cost of investment was included; therefore, any positive number should be interpreted as a positive ROI.

Data analysis was performed on countries with sufficient volume and quality of input data (greater than 10 services utilised per country), a total of 57 countries. As the Europe and North American regions launched in 2018, comprehensive data were not available and were not included in this analysis. HIV treatment service was not included as data were of insufficient quality to analyse.

## Results

P4P has fully launched in 130 countries as of 2018, with an initial pilot and annual deployment based on a regional approach that addressed the countries with more significant needs first and developed countries later. The programme covers 235,482 total persons, including 100,430 employees and 165,120 dependents. Total utilisation of the programme at time of analysis includes 124,839 individual services delivered. Where the programme has been in place for the full launch and 24-month sustainment phase, it was utilised by approximately 45.9% of covered persons. Utilisation and ROI by service category are detailed in Table 1; immunisations made up a large portion of service availability and utilisation. Immunisations, cardiovascular health, and smoking cessation are estimated to yield the highest returns among interventions. Global and regional ROI are detailed in Table 2, and individual country ROI are detailed in Table 3. Regional, global, and service category ROI were calculated using a population-weighted average of individual country and service ROI.

**Table 1:** Programme Utilisation and ROI (USD) by Service Category

Service	Total ROI	Utilisation	# Countries
Adult Vaccine	5.35–17.88	54,262	112
Child Vaccine	6.16–18.09	24,262	99
Preventive Examinations	(1.00)–(1.00)	20,646	74
Cardiovascular Health	47.97–79.66	14,753	73
Cancer Screenings	(1.42)–(1.41)	4930	100
Prenatal	(0.85)–(0.81)	3876	58
Tobacco Cessation	15.91–27.96	242	78
HIV	N/A	431	27
Other Related Services	N/A	1627	N/A
Total		124,839

**Table 2:** Programme Demographics and ROI (USD) by Region

Region	Total ROI	Total Participants	Employees	Dependents	Launch
PILOT	11.29–24.59	2710	744	1966	2012
MENE	7.58–14.20	8202	2767	5565	2013
LATAM	3.02–14.70	16,099	5346	10,753	2014
AF-PAK	7.58–14.20	18,078	5366	12,712	2015
R-ISB	1.85–6.36	45,402	12,243	26,280	2016
ASIA-PAC	4.38–12.52	33,004	14,409	18,595	2017
Total	4.28–11.81	116,614	40,875	75,871

**Table 3:** Programme Demographics and ROI (USD) by Country

	Country	Total ROI	Participants
PILOT	Nigeria	21.03–45.15	1192
	Romania	0.78–2.59	1085
	Ecuador	4.84–10.64	361
	Ghana	40.67–85.84	72
AF-PAK	Pakistan	15.96–27.90	8536
	South Africa	1.07–6.11	1541
	Algeria	(1.58)–(1.58)	1272
	Kenya	2.12–10.17	987
	Morocco	(4.50)–(4.49)	417
	Tunisia	(3.65)–(3.60)	116
	Ethiopia	17.52–43.00	68
	Uganda	4.31–11.68	55
	Rwanda	(5.61)–(5.61)	27
LATAM	Brazil	4.10–18.42	5396
	Argentina	5.00–21.57	4108
	Mexico	(0.45) – 1.51	2442
	Costa Rica	(0.79)–(0.44)	1305
	Colombia	9.86–45.02	936
	Chile	(0.92)–(0.23)	661
	Panama	(1.05)–(1.04)	501
	Peru	0.96–9.19	401
	Dominican Republic	(2.31)–(2.31)	110
	El Salvador	(1.17)–(0.92)	67
	Honduras	(0.66) – 0.63	66
	Jamaica	1.44–10.01	56
	Venezuela	(1.88) – 0.48	50
MENE	Egypt	9.72–22.72	5079
	Turkey	2.34–6.28	1871
	UAE	8.68–29.84	923
	Lebanon	6.29–15.58	203
	Kuwait	57.61–159.60	108
	Yemen	14.30–31.36	83
RISB	India	1.29–5.59	30,869
	Russia	(0.06) – 2.51	2604
	Bangladesh	3.55–8.58	2305
	Sri Lanka	9.00–16.28	1295
	Ukraine	10.85–22.65	910
	Kazakhstan	(1.41)–(1.39)	216
	Belarus	3.55–6.02	115
	Georgia	22.25–41.47	68
	Azerbaijan	1.39–5.55	66
	Moldova	(0.23) – 2.73	37
	Uzbekistan	9.39–18.60	36
ASIA-PAC	China	1.69–8.73	10,295
	Japan	9.29–21.08	7040
	Singapore	3.29–12.63	4730
	Indonesia	6.11–10.08	2995
	Malaysia	3.44–6.33	2013
	Korea	0.89–3.30	1652
	Philippines	10.76–25.69	1304
	Vietnam	4.49–18.98	1143
	Taiwan	2.99–9.84	567
	Thailand	6.32–17.47	477
	Hong Kong	(11.76)–(11.76)	415
	Cambodia	2.03–7.71	182
	Myanmar	2.02–5.48	118
	New Zealand	0.10–9.28	73

The overall global ROI of P4P, based on two years of programme activity, was estimated between $4.28–$11.88, demonstrating a significant ROI for the programme over a five-year period. This estimated ROI measurement was positive for most and very significant for many countries. Key inputs drive the ROI results to the benefit and cost estimates.

Costs vary primarily by 1) price of services and 2) programme implementation details, such as administrator or local market factors. For example, some markets had more competitive bids which better controlled costs, others had fewer options or required higher cost solutions. Benefits differ fundamentally by 1) programme design or which specific service offerings are available in each country, 2) disease burden (morbidity and mortality), 3) utilisation of services, and 4) country population. For example, a country with more gaps in higher ROI services and utilisation (such as vaccines) and/or a large burden of disease will have a much higher ROI than another country with gaps in lower ROI services (such as screenings) or with a low burden of disease. However, despite inter-country input variation, all overall regions and nearly all large population countries have a positive programme ROI.

## Discussion

In the context of business investment, this is a significant result that is not easily replicated through alternative investment options. The ROI calculation is also likely an underestimate of the actual return to company, given potential longitudinal and less tangible program benefits. P4P is a novel programme, and there are difficulties in developing a model with limited prior literature evidence. The published RAND Europe report discusses this, stating the model was designed to provide a conservative estimate of ROI, taking lower bound estimates of benefits and upper bound estimates of cost. The cost modelling of five years may also minimise more long-term benefits. The resulting ROI estimate is therefore “likely to be lower than the actual return”, per the RAND Europe report.

Beyond this structural design of the model, there are also other benefits that the programme may accrue that are not measured. This analysis only provides a return on services paid for by the programme. P4P promotes all 40 preventive services in the portfolio, but only pays for those which are not already covered. By increasing awareness of health prevention, the programme aims to increase utilisation of all 40 services. Many services already provided by governments and health plans, such as some child immunisations, are among the lowest cost and highest benefit, with P4P paying for others with a less robust ROI profile. Therefore, there is an additional potential benefit to the company of these other promoted services.

The ROI estimates also only apply to the company, and although not included in the model, the programme may have broader economic and health-related benefits to participants. These broader benefits are particularly relevant for children, prenatal, and other interventions which primarily benefit dependents, but have a somewhat limited direct return to the company. Interventions are also based on the evidence as assessed by RAND Europe, and some of these, such as preventive examinations, have limited evidence for ROI in isolation but are part of an integrated effort by P4P to drive awareness and utilisation of other services. The positive externalities generated by these aspects of the programme (i.e., those with lower company-attributable ROI) may translate to additional tangible or intangible benefits, such as increased preventive health activities or organisation reputational value.

Some limitations also exist in this analysis. For country-specific inputs where specific data were not available, evidence-based assumptions informed best estimates for the model. For example, valid immunisation rates are not available for all countries, but the best available information was utilised for either the country or region. While data quality was generally very consistent, there was some variation that limited analyses as previously mentioned. Also, the model underlying the analysis was based on existing evidence for each covered service, which varied in quality and relevance to this programme.

The programme also contains a comprehensive and multidisciplinary local team and communication strategy to enhance awareness of services and address cultural barriers. This strategy is another critical aspect of the programme, as service availability must be accompanied by engagement mechanisms to drive utilisation and result in a positive return. Engaging critical stakeholders from business functions enables the programme to function sustainably and deliver on its value proposition. It is also important to emphasize that while the positive ROI is encouraging and provides further programme validation, it is only one measure of success and was not the primary objective of P4P when the programme was initiated. This may be of relevance to organisations considering similar or related programmes to enable initial buy-in and set appropriate expectations.

Overall, P4P delivers expanded access to and utilisation of preventive health services by employees and their families, resulting in a significant positive ROI to the company. This study can supplement existing health and wellbeing evidence, and strongly supports a renewed focus for multinational companies to focus on addressing disparities in access to essential clinical preventive services. The programme also has a positive value proposition to maintain a motivated and healthy workforce, reduce the cost of ill health, provide a competitive advantage for recruitment and retention, and drive the adoption of a culture of health.

## Conclusion

The P4P programme was successful at producing utilisation of clinical preventive services and the model estimates delivering a significant positive ROI to the company. Furthermore, these numbers likely underestimate the actual return due to use of conservative modelling and generation of benefits not included in the model. This provides a strong incentive for multinational companies to focus on clinical preventive services to supplement or focus on existing health and wellbeing programmes.

## Data Availability

The data that supports the findings of this study are available from GlaxoSmithKline (GSK), but restrictions apply to the availability of these data, and so are not publicly available. Data are however available from the authors upon reasonable request and with permission of GlaxoSmithKline (GSK).

## Abbreviations

P4P: Partnership for Prevention
ROI: return on investment
